# Monthly Summary

**Published:** 1894-05

**Authors:** 


### Monthly Summary.

SPONGY GUMS.—Spongy and swollen gums, a condition often attendant upon middle age, may or may not be the result of any particular disease; but they are very inconvenient, and may even cause serious trouble.

In their healthy state the gums are firm and, it may be, somewhat hardened, with just enough blood present to color them a delicate pink. Gums in this condition offer a support to the teeth which it would be hard to surpass.

In the disordered condition of which we are speaking, however, they become swollen, and are so discharged with blood as to present an appearance of having been parboiled. The slightest disturbance is sufficient to cause a flow of blood, while there is a constant sense of discomfort, and a constant desire to pick at or suck them.

Soon the teeth become more or less loosened, and by reason of the pressure of the tongue and the food behind them, tend to spread apart and protrude outward. The substance of the tooth is next attacked, and the tooth becomes discolored and decayed. The gums refuse longer to hold the teeth and, in fact, time alone is necessary to convert the whole mouth into a useless and disgusting object.

As we have seen, all this may be consequent upon an over-supply of blood to the gums. The remedy is rather preventive than curative.

First of all, we have to consult with the family physician to learn whether or not the system needs "toning up," as is not unlikely to be the case. Probably he will prescribe also some astringent mouth wash.

But whatever may be the result of our consultation with the family doctor, we must at once begin a systematic "exercise" of the gums, and continue it every night and morning. A tooth-brush must be selected more for its stiffness than anything else, and with a little cool water and castile soap, or even cold water alone, we must literally scrub the gums, paying heed to neither blood nor feelings until we are satisfied that we have eradicated all traces of stagnant blood from the porous tissues.

This may seem rather harsh treatment, but if we persist in it we shall be rewarded.

It would be difficult to overestimate the influence which a healthy gum may exert over the teeth.—*Youth's Companion.*

---

HOW AN OLD STORY ORIGINATED.—"It is singular," said E. S. Vickers of Richmond, Va., who was shaking hands with a number of old acquaintances in the city yesterday, and who registered at the Southern Hotel, "how many times and in how many different ways a good story gets told. Everyone has heard about lawyers and doctors charging $100 for an opinion, and then explaining that the charge is $1 for the service rendered and $99 for knowing how to render it. I believe all these stories originated from James Alfred Jones, a well-known attorney of our city, who, years ago, was looked upon as one of the ablest lawyers in the country, and certainly in the South.

On one occasion he was consulted by a very wealthy, close-fisted old gentleman, who asked his opinion on a point of law. Mr. Jones listened to the statement, and then wrote an opinion on a single sheet of legal cap, which he handed over to his wealthy friend. The latter, after glancing through it, put his hand in his pocket, and, bringing out two or three bills of small denominations, asked the usual question. When the lawyer said his charge was $500, the seeker after legal lore made use of some remarks unfit for publication and reminded his friend that the whole business had not occupied more than 15 minutes.

Mr. Jones took off his spectacles, wiped them deliberately, and, pointing to his immense library of books, asked if his client would like to read them all carefully for $500. The client said of course not. "Well, then," retorted the lawyer, what are you grumbling about? I had to read every one of those books once, and a good many of them twice, before I was able to give you the opinion you wanted, and it is none of your business whether I read them before you came in or after. If I made you wait while I read them you would have thought my fee well earned, but I should have caused you to lose time worth a great deal more than $500 to you while I was doing it."—*St. Louis Globe-Democrat.*

---

ELECTRICITY IN 1900.—*The Sun's* special dispatch from New York gives a hint of the many things electricity may do for us in the next six years. Already messages are being printed by telegraph at a rate of forty words a minute on pages similar to ordinary typewritten sheets. The overhead trollery is to give way, it is said, to an underground substitute, and in town and country electricity will be the favorite means of traction. Power will be sent over wires for manufacturing and domestic purposes. Electric heating will come to stay, displacing the troublesome range, stove and furnace. The head of the house will not need to rise cold, frosty mornings to bring up coal and spoil his temper, but will lie abed, after pressing a button, till his room is warm, and then go down to a hot breakfast cooked by electricity. By 1900 telephone messages may perhaps be sent across the Atlantic. The new applications of this mysterious force are likely to exceed all present computation. Even seeing by electricity is hinted at as a possibility.

---

TRICHLORACETIC ACID is having such success that I must say a word about it. Where you have a spongy condition of the gums you desire to remove, or a growth over a third molar, or any condition of that kind, it is the most appropriate application that can be made, by simply taking a little wooden spatula, saturating it, and rubbing it over the tissue. It is a powerful escharotic, and in one or two applications you will remove the abnormal growth. Again, where you have the little nodules of calcific deposit on the roots of the teeth, with the same little wooden spatula you pass down into the apex and cleanse the surface of these roots. Wherever we have calcific deposits, we use this acid, even in its full strength, in that way. It cleans the root thoroughly. After you have used your scalers, use this, and it will remove every vestige of the calcic deposit. Then, again, it has a very happy result on the tissues themselves. Being a powerful escharotic and astringent, you will find that an application into the pocket will arrest the accumulation of pus that is so common, with one or two applications. I have had very desirable results from its careful use in pyorrhea. If I open into the root of a tooth, I dress the pulp by taking a little circular spatula and forcing it up into the root; the acid will destroy the tissue and purify the root in a moment, more perfectly than you can with carbolic acid.—Dr. Peirce, in *International.*

---

EFFECTS OF PREGNANCY.—All dentists have observed the deterioration of the teeth during child-bearing. Why is it ? Some say it is because the growth of the child exhausts the vitality of the mother. Some contend it is because of the abnormal activity of the vital processes. It is certainly not because of the mother's condition *per se*, or we should find it more usually prevailing during this condition, whereas it is only seldom. We believe it is generally from the sympathetic activity of the urinary organs producing an excess of uric acid throughout the system. We believe we have almost invariably found this in these sudden attacks on the teeth, without reference to pregnancy. The reason we find it more frequent during pregnancy is because they are more likely to be irritated.—ED., *Items of Interest.*

PUTRESCENT PULPS.—There is still a want of unanimity in treating putrescent pulps, but there is an evident advancement in our knowledge and skill. Many more dead teeth are saved than formerly, and some dentists are notably successful. Isn't it strange that some dentists utterly fail with apparently the same treatment that give others eminent success? It seems a very simple thing with some; with others it is impossible.

"Every one has his hobby," says one confused with his many failures in many methods. But, my dear sir, there are not "so many methods." Perhaps we might include all under two—the use of coagulants and of anti-coagulants. One man will tell you, and he may perhaps be considered of the old school, that there is nothing like carbolic acid and creosote. The very fact that these are coagulates pleases him. Another successful practitioner will aver with Dr. Swift, of New York, "I have long since discarded coagulants; they dam up the tubuli, preventing disinfection and diffusive action. I employ diffusible essential oils, such as eucalyptus, eugenal, oil of cassia, and myrtol, which are very diffusible, carrying a large quantity of oxigen and depositing volatile camphors, which are all powerful in the destruction of septic and infectious matter." This may be considered the new school of practice. The main thing is to be successful, but whichever method is used, if a few can succeed, why not more?—ED., *Items of Interest.*

---

OF what use is it to be rich and honored, if not healthy and happy? Yet how often we sacrifice health and happiness for wealth and glory. We make life a continual strife for fame and possessions at the expense of every comfort, imagining that by and by we will rest on our laurels and enjoy ourselves. But after our whirl of work and fret, we are unfitted for either rest or enjoyment, and enter prematurely into old age and decay. We become so habituated to hurry and worry, and the ambition for gain, we cannot shake them off when weakness and infirmities necessitate retirement and rest.—*Items of Interest.*

BACTERIOLOGY.—Recent studies in bacteriology have not been carried on in the line which the original investigators had laid down. We have recently discovered bugs in everything, and if the conditions of things are such as we are now taught, we would have been permeated with disease and the whole world have been dead long ago. I do not wish to be understood as saying that bacteria does not play an important part in many things, but we have gone to extremes. I am willing to go on record as saying that acid plays a far greater part in causing decay than bacteria, and that the location of decay in the smaller cavities of the teeth is only the indigenous home of bacteria. *Dr. E. S. Chisholm.*

---

TO CUT OFF CROWNS.—To cut off a tooth, drill directly through it with a small size spear point drill, being careful that the drill comes out on the lingual surface of the tooth at the same distance from the gum as where it entered. Now take the next size larger, drill and enlarge the hole already made, and so on till the tooth is entirely cut off. The end of the root will be left perfectly smooth and ready for the crown. This method does away with the disagreeable jar, noise and slipping which is always complained of by the patient where a bur or facer is ured.—*Office and Laboratory.*

---

TESTING GOLD AND SILVER.—Use pure nitric acid. To test: File the metal clean to make sure that you are testing the metal itself and not a plating or covering of any sort. Silver under the action of the acid goes to a peculiar grey color. If brass it will turn green. German silver will do the same; nickel will go black. Gold, pure, 22k. or 18k., will be unaffected, and the acid will stand like water; 15k. will turn very slightly brown if the acid is pressed well in with the bare finger; 12k. will go the same without any pressing in; 9k. goes a decided brown at once.—*English Mechanic*

